# Effects of interactions between facial expressions and self-focused attention on emotion

**DOI:** 10.1371/journal.pone.0261666

**Published:** 2021-12-23

**Authors:** Ryota Kobai, Hiroki Murakami

**Affiliations:** 1 Department of Psychology, Oita University, Oita, Oita, Japan; 2 Department of Sleep-Wake Disorders, National Institute of Mental Health, National Center of Neurology and Psychiatry, Kodaira, Tokyo, Japan; Aristotle University of Thessaloniki, GREECE

## Abstract

Self-focus is a type of cognitive processing that maintains negative emotions. Moreover, bodily feedback is also essential for maintaining emotions. This study investigated the effect of interactions between self-focused attention and facial expressions on emotions. The results indicated that control facial expression manipulation after self-focus reduced happiness scores. On the contrary, the smiling facial expression manipulation after self-focus increased happiness scores marginally. However, facial expressions did not affect positive emotions after the other-focus manipulation. These findings suggest that self-focus plays a pivotal role in facial expressions’ effect on positive emotions. However, self-focusing is insufficient for decreasing positive emotions, and the interaction between self-focus and facial expressions is crucial for developing positive emotions.

## Introduction

Self-focusing is a cognitive activity that modulates emotional states. Moreover, self-focused attention is defined as an awareness of self-referent, internally generated information that stands in contrast to an awareness of externally generated information derived through sensory receptors [1]. Numerous studies have suggested that self-focus is related to negative emotions [2] and clinical disorders [1]. Ingram [1], in a review of self-focus and psychopathology, suggested that self-focus is associated with depression, and several psychopathologies, including anxiety, substance abuse, schizophrenia, and psychopathy. The degree of private self-consciousness, which is defined as the tendency to attend to one’s feelings and thoughts, was associated with state-anxiety, trait-anxiety, and worry in a healthy population [3]. Moreover, case studies of anxiety disorder patients have indicated that procedures evoking external attentional focus by shifting attention away from self-focus improve symptoms [4, 5].

Associations between negative affect and self-focused attention have been reported not only in studies using questionnaires and clinical case studies but also in experimental laboratory studies [6, 7]. Pyszczynski et al. [7] indicated that depressed participants in a self-focus condition manipulated by writing stories using self-referent word lists showed more pessimistic thoughts than externally focused depressed individuals. Also, Nix et al. [6] conducted the same manipulation as Pyszczynski et al. [7] with depressed individuals and demonstrated that participants in a self-focused manipulation showed higher depressed mood than participants in an externally focused condition writing stories using another-referent list. Moreover, a recent study investigated the effect of an attention training technique. The study demonstrated that attention training reduced self-focused attention and cognitive anxiety of individuals with a high self-focus before the manipulation, compared to a distraction method in which participants engaged in guided imagery of neutral events while listening to classical music in the background [8].

Interacting Cognitive Subsystems (ICS) is a theoretical framework that accounts for all information-processing aspects [9]. In this framework, self-focus is described as cognitive processing for maintaining depressive states [10]. Similar to cognitive processing, bodily feedback such as frowning expressions also perpetuate the depressive configuration [11]. ICS analysis also suggests that emotional response can be altered by sensory feedback such as smiling facial expression [12]. Yamamoto, Sugimori, and Shimada [13] investigated the effect of facial expressions after self-focused attention. They reported that negative mood decreased in the smiling expression condition compared to the control expression condition which participants were instructed to purse their lips lightly after self-focused attention, and positive mood decreased in the control expression condition compared to the smiling expression condition after self-focused attention. Their study, however, did not examine the effect of facial expressions without self-focused attention. Therefore, the interaction between facial expressions and self-focused attention remains unclear.

The current study was designed to investigate the effects of the interaction between facial expressions and self-focused attention on emotions by including an other-focus condition. The present study was expected to replicate the study results by Yamamoto et al. [13] regardless of the self-focus manipulation, and self-focus would enhance the effect of facial expressions on positive and negative emotions. Furthermore, Nolen-Hoeksema [14] argued individual differences in the effect of self-focus. She suggested that a ruminative response style might have adverse effects only on pessimists and not on optimists. Therefore, the present study also investigated individual differences in anxiety on the interaction between facial expressions and self-focused attention. We hypothesized that negative emotions would be more robust in high anxious individuals under self-focus.

## Methods

### Participants

Undergraduate volunteers (N = 64, 33 men and 31 women, mean age 21.1 years, SD = ±1.1) recruited from Oita University participated in this study. The participants were randomly allocated to each condition in a 2 (focus: self-focus vs. other-focus) × 2 (facial expression: smile vs. control) design.

All the participants provided written informed consent before participating in the study following the Helsinki Declaration. This study was approved by the Ethics Committee of the Faculty of welfare and health science of Oita University. The approval number is 36.

### Measures

The Trait form of the validated Japanese version [15] of the State-Trait Anxiety Inventory (STAI-T; [16]) was used to assess participants’ trait anxiety. The emotional state assessment [17] consisting of 10 positive emotional items and ten negative emotional items. Three of each (positive: happy, good mood, light-hearted; negative: pessimistic, feel inferior, lost confidence) was selected from the original assessment for this study.

### Procedure

The study was conducted in a laboratory. The participants entered the laboratory and first gave their written informed consent for participating in the study. Then, they were seated and they completed the STAI-T and the emotional state assessment for assessing their baseline emotional state, which was followed by the attention manipulation. The participants in the self-focus condition were asked to respond how 92 personality trait-words described them, which was rated on a 7-point scale ranging from “not characteristic for me” to “extremely characteristic for me.” Then, they were asked to complete ten sentences following the phrase, “I am …” These self-focus manipulations were similar to those in the previous study by Sakamoto [17]. Unlike the previous study, however, the other-focus condition participants also completed ten sentences that followed the phrase, “Prime Minister Abe is …”.

After the attention manipulation, the facial expression manipulation (smile vs. control), which was similar to that in previous studies [13, 18, 19], was conducted for 15 seconds. In the smile condition, the participants were instructed to draw the corners of their mouth back and up [13, 18]. In the control condition, the participants were instructed to purse their lips lightly [13]. The emotional state scores were assessed after all the manipulations were completed. After the experiment, the participants were asked whether they were aware of the aim of the facial expression manipulation.

### Data analysis

An analysis of variance (ANOVAs) analyzed emotional state scores based on the attention (self-focus vs. other-focus) and facial expression conditions (smile vs. control) as between-subject factors and time (pre vs. post) as a within-subjects factor. Ryan’s method (p < .10) was used for post hoc analyses of significant or marginally significant interactions to identify significant differences in data point combinations. We calculated partial eta squared (*η*^*2*^_*p*_) as an index of each ANOVA’s effect size. Also, Pearson correlation coefficients were computed between STAI-T scores and post-manipulation difference scores compared to pre-manipulation baseline emotional state scores in each condition to examine relationships between the conditions and trait anxiety.

## Results

**Fig 1:**
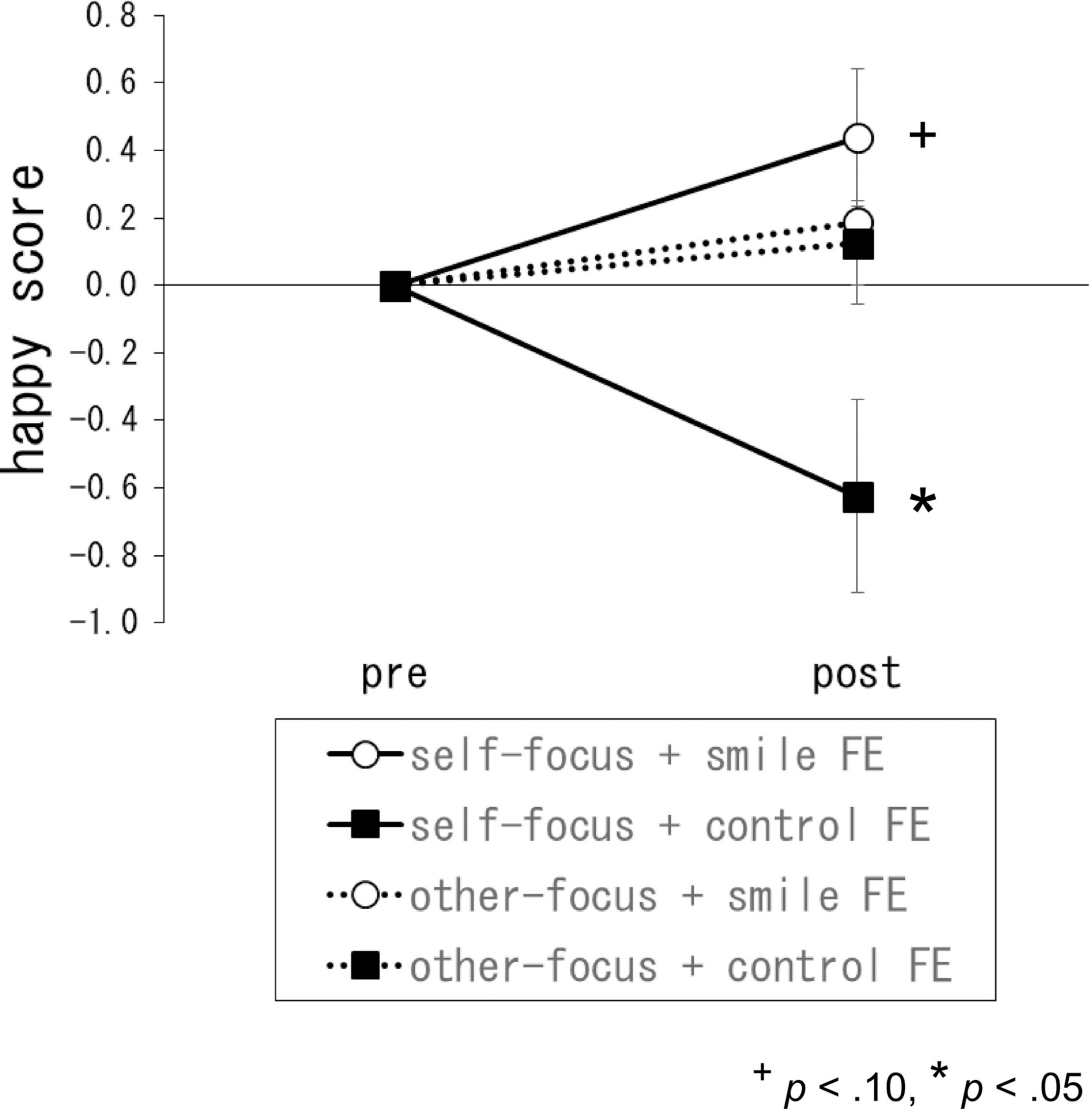
Change scores of the emotional state of happiness for each condition. Error bars indicate standard errors of the mean. FE = facial expression.

No participants were aware of the purpose of the facial expression manipulation. The means and standard errors of the emotional state scores in each condition are shown in Table 1. An ANOVA conducted on happy scores showed a significant interaction between facial expression and time, *F* (1, 60) = 6.35, *p* < .05, *η*^*2*^_*p*_ = .10 (Fig 1). Ryan’s test indicated that the happy score was marginally higher in the smile condition (*p* < .10). Moreover, the interaction of the happy score between the three factors (attention condition, facial expression, and time) was significant, *F* (1, 60) = 5.01, *p* < .05, *η*^*2*^_*p*_ = .08. Ryan’s test indicated that the control facial expression manipulation after the self-focus manipulation significantly decreased the happy score (*p* < .05), whereas the smiling facial expression after the self-focus manipulation marginally increased the happy score (*p* < .10).

**Table 1:** Means and standard errors of emotional state scores.

	self-focus				other-focus			
	smile FE		control FE		smile FE		control FE	
emotional state	pre	post	pre	post	pre	post	pre	Post
happiness	5.50	5.94	5.69	5.06	5.25	5.44	5.44	5.56
	(0.29)	(0.27)	(0.27)	(0.35)	(0.35)	(0.22)	(0.20)	(0.20)
good mood	5.50	5.56	5.31	5.13	5.31	5.19	5.19	5.31
	(0.26)	(0.29)	(0.28)	(0.27)	(0.28)	(0.26)	(0.36)	(0.28)
pessimistic	2.69	2.63	2.75	2.31	2.31	1.94	2.88	2.31
	(0.37)	(0.45)	(0.42)	(0.39)	(0.36)	(0.23)	(0.46)	(0.31)
feel inferior	3.13	2.81	2.81	2.44	2.81	2.38	2.94	2.63
	(0.46)	(0.37)	(0.45)	(0.40)	(0.39)	(0.33)	(0.46)	(0.38)
light-hearted	4.75	5.06	4.81	4.88	0.33	5.06	5.00	4.81
	(0.21)	(0.28)	(0.26)	(0.26)	(0.26)	(0.27)	(0.29)	(0.28)
lost confidence	2.88	2.44	2.88	2.44	2.81	2.25	3.50	2.75
	(0.34)	(0.33)	(0.38)	(0.38)	(0.38)	(0.34)	(0.43)	(0.44)

*Note*. Standard errors are shown in parentheses. FE = facial expression.

Furthermore, the main effect of time was significant for all the negative scores, pessimistic, *F* (1, 60) = 5.87, *p* < .05, *η*^*2*^_*p*_ = .09, feel inferior, *F* (1, 60) = 7.68, *p* < .01, *η*^*2*^_*p*_ = .11, and lost confidence, *F* (1, 60) = 16.69, *p* < .0005, *η*^*2*^_*p*_ = .22, with all the negative states decreasing after the manipulation. There were no other significant differences in emotional state, *Fs* < 1.26, *p* > .26, *η*^*2*^_*p*_ < .02.

The Pearson correlation coefficients between the STAI-T scores and post manipulation scores compared to the pre-manipulation baseline emotional state scores in each condition are shown in Table 2. It can be seen that there were no significant differences between post manipulation and pre-manipulation scores, *rs* < |.46|, *ns*.

**Table 2:** Pearson correlation coefficients between the STAI-T scores and the difference scores of the post manipulations compared with before manipulations baseline of the emotional state scores for each condition.

	self-focus		other-focus	
emotional state	smile FE	control FE	smile FE	control FE
happiness	0.15	0.03	0.14	0.06
good mood	0.12	0.20	-0.29	0.29
pessimistic	0.10	-0.21	0.14	-0.46
feel inferior	0.17	0.22	0.07	-0.39
light-hearted	0.37	0.17	-0.05	0.10
lost confidence	-0.13	0.27	0.29	-0.19

*Note*. FE = facial expression.

## Discussion

This study was designed to investigate the interaction between facial expressions and self-focused attention on emotion. This interaction’s effect on positive emotions in this study partially replicated the findings of a previous study by Yamamoto et al. [13]. This study demonstrated that a control facial expression manipulation after self-focused attention reduced happiness, whereas a smiling facial expression marginally increased happiness. However, facial expressions did not affect positive emotions after other-focused attention. These findings suggest that self-focus plays a pivotal role in the effect of facial expression on positive emotions. Additionally, self-focus alone is not sufficient for decreasing positive emotions. The interaction between self-focus and facial expression is essential for affecting positive emotions.

This study investigated the interaction between facial expressions and self-focused attention on the emotions of healthy people, because previous studies have investigated that such interactions are observed in clinical populations as well as in healthy populations [13, 20]. However, the self-focus manipulation did not influence negative emotions regardless of facial expressions, which failed to support this study’s hypothesis. Nolen-Hoeksema [14] suggested that the effect of self-focus depends on individual differences. In the present study, the participants were not clinical patients. Previous studies have indicated that self-focus manipulation is effective for highly depressed and highly anxious people’s negative emotions [21, 22]. In this study, we conducted a correlation analyses between the effects of the manipulations and participants’ trait-anxiety. However, we might not have detected any individual differences because of the small sample size. It is suggested that the interaction between facial expressions and self-focused attention is investigated by considering individual differences, including clinical populations and more sample size.

## Supporting information

S1 Dataset (PDF)
