# Supplementary material for: Effects of interactions between facial expressions and self-focused attention on emotion
Source: PLoS One. 2021 Dec 23;16(12):e0261666. doi: 10.1371/journal.pone.0261666 (PMC8699986; doi:10.1371/journal.pone.0261666)
Supplement: S1 Dataset — (PDF) [file pone.0261666.s001.pdf]

		happiness		good mood		pesimistic		feel inferior		light-hearted		lost confidence		STAI-T
		pre	post	pre	post	pre	post	pre	post	pre	post	pre	post	
self-focus	smile FE	5	6	5	7	2	1	1	2	4	5	2	1	38
		7	7	6	6	4	2	2	2	5	5	3	2	37
		4	3	7	7	5	1	6	3	5	7	5	2	53
		6	6	4	4	5	7	7	6	4	4	4	4	42
		5	7	5	6	1	1	1	1	6	6	1	1	40
		3	5	6	6	5	5	3	4	4	5	4	3	59
		6	6	6	4	2	2	2	1	5	3	3	1	34
		7	7	6	5	3	2	5	4	5	5	3	3	28
		5	6	3	3	4	5	5	5	3	4	5	5	52
		6	6	5	6	1	2	4	3	6	6	2	4	38
		6	6	6	6	2	3	2	3	4	5	4	3	46
		6	7	5	5	3	3	3	2	4	4	2	2	40
		7	7	5	5	2	2	4	2	5	6	2	2	52
		4	5	6	6	2	4	2	4	5	4	4	4	53
		5	5	6	6	1	1	1	1	6	5	1	1	53
		6	6	7	7	1	1	2	2	5	7	1	1	48
	control FE	7	7	5	7	3	1	3	1	5	7	3	1	45
		6	4	4	4	5	5	4	4	4	4	4	4	46
		6	6	6	5	2	2	2	2	5	5	2	2	42
		6	6	4	5	5	3	5	3	3	4	4	2	35
		5	5	7	6	5	5	7	5	6	6	5	5	40
		6	3	6	6	3	2	2	1	6	6	1	1	36
		5	5	6	3	1	1	1	1	6	3	1	2	40
		5	6	3	4	5	4	5	5	4	4	5	4	46
		3	2	6	6	2	1	1	1	5	5	1	1	64
		6	6	4	5	1	1	1	1	4	4	3	3	40
		6	5	6	5	2	2	3	3	5	5	4	4	25
		7	7	5	4	1	1	1	1	4	4	2	1	31
		4	4	7	7	1	1	4	3	6	5	5	2	34
		6	5	6	5	1	1	1	1	6	6	1	1	45
		6	6	5	5	5	5	3	5	3	5	3	5	47
		7	4	5	5	2	2	2	2	5	5	2	1	39
other-focus	smile FE	4	4	6	4	3	2	5	5	6	5	4	4	44
		7	6	5	5	1	1	2	2	6	5	1	1	36
		4	5	3	3	5	3	5	2	4	3	5	5	40
		7	7	5	5	3	2	5	4	2	3	5	2	31
		3	5	6	6	2	2	2	2	5	5	2	3	56
		5	5	6	5	2	2	3	3	5	5	3	3	46
		5	6	5	6	1	1	1	1	5	5	2	1	32
		5	5	4	5	1	1	3	1	6	5	4	1	46
		3	5	6	6	5	3	5	4	5	5	5	3	44
		4	4	7	6	3	2	2	2	5	5	1	1	37
		6	6	5	4	1	1	1	1	6	7	2	1	48
		5	5	4	4	4	4	4	4	5	5	4	4	49
		7	7	4	6	1	1	1	1	4	6	1	1	34
		6	6	6	6	1	2	2	2	5	5	2	2	40
		7	5	7	7	1	1	1	1	5	7	1	1	48
		6	6	6	5	3	3	3	3	6	5	3	3	40
	control FE	5	6	7	7	3	3	3	3	3	3	3	3	39
		6	6	5	6	6	3	6	4	4	5	6	6	43
		6	6	3	3	1	1	1	1	5	5	1	1	50
		5	6	6	7	2	1	2	1	5	6	3	1	51
		4	4	3	5	5	3	5	4	4	4	5	4	51
		6	6	6	5	2	1	2	3	4	4	5	5	26
		5	6	4	4	2	3	2	3	4	4	5	3	37
		5	5	5	6	2	4	3	4	5	4	4	4	31
		6	6	4	4	6	5	6	5	4	4	6	5	37
		4	4	3	4	4	2	5	5	6	6	2	1	45
		6	5	5	6	4	3	4	3	5	5	5	3	40
		6	6	7	5	1	1	1	1	6	4	4	4	45
		5	5	6	6	5	2	1	1	7	6	2	1	51
		5	5	6	6	1	1	1	1	6	6	1	1	45
		6	6	7	5	1	3	4	2	5	4	2	1	39
		7	7	6	6	1	1	1	1	7	7	2	1	38
